# Metrological Timelines in Traceability

**DOI:** 10.6028/jres.103.005

**Published:** 1998-02-01

**Authors:** Charles D. Ehrlich, Stanley D. Rasberry

**Affiliations:** National Institute of Standards and Technology, Gaithersburg, MD 20899-0001

**Keywords:** calibration, equivalence, measurement assurance, measurement comparisons, measurement uncertainty, metrology, timelines, traceability, traceability statements

## Abstract

There is a growing requirement for an internationally accepted system of recognition of measurement capabilities and relationships within and among countries, to facilitate seamless global commerce and trade. As a result, metrologists worldwide have recently developed increased interest in the concept and definition of traceability. Classically, traceability provides a way of relating the results of a measurement (or value of a standard) to higher level standards. Such standards are usually national or international standards, and the comparisons used to provide the traceability must have well-understood uncertainties. An additional complexity arises because all instruments and standards are subject to change, however slight, over time. This paper develops approaches for dealing with the effects of such time-dependent changes as a part of traceability statements. The use of metrological time-lines provides a means of effectively visualizing these relationships in a statement of traceability. When the rate of change in the measurement process is sufficiently small, the approach proposed here is less important. However, documented measurement assurance procedures are required at all levels so that appropriate uncertainties may be estimated with confidence. When laboratory or national boundaries are crossed in the traceability process, other factors come into play, and the original concept of traceability can become obscure. It is becoming common to hear the term “equivalence” used to describe these more complex measurement relationships.

## 1. Introduction

Worldwide commerce requires a coherent measurement system within which the consistency of measurements is easily maintained and demonstrated. Buyers and sellers needed such a system in order to evolve from barter to patterns of trade which use specifications to describe such things as size or performance. Classically, traceability [1] provides a way of relating the results of a measurement (or value of a standard) to higher level standards. Such standards are usually national or international standards, and the comparisons used to provide the traceability must have well-understood uncertainties. There is growing interest in the practical use of traceability to demonstrate the integrity of comparisons, and for that matter, to define just what it is that is being compared. Since all instruments and standards are subject to changes, however slight, over time, the use of metrological timelines (described below) greatly facilitates the visualization of measurement relationships in a statement of traceability. This paper discusses these issues, and addresses the need for new terms to describe the concepts usually associated with traceability.

### 1.1 Requirements for Traceability

Depending on the measurement requirements and the resources available, the need for traceability and the form it takes may vary considerably. Manufacturers want the benefits of traceability so that customers will know the available level of performance for instruments and materials. In government applications, regulators may demand traceability to help ensure that public safety requirements are met. Further, armed services use traceability to provide a coherent measurement system for protecting lives, including those of the servicepeople. Traceability rarely stands alone; rather it is a part of larger systems which call for such properties as interchangeability of manufactured parts, quality systems in production of pharmaceuticals, and safety in air traffic control systems.

Common to all of these requirements is a need to know the results and uncertainties of measurements: better and more compatible products and services are produced throughout society when measurement variability is reduced. This in turn leads to more equitable trade and more efficient economies.

It is typically the responsibility of a national metrology institute (NMI) to provide its nation’s measurement infrastructure with access to accurate measurement capability. The comparability of measurements and associated uncertainties with those of other nations is determined through a variety of mechanisms, including bilateral comparisons and round robins of international measurements. As discussed below, such comparability does not necessarily constitute traceability [2].

Requirements for measurement accuracy translate into a need to know not only the results of measurements but the uncertainties associated with the results. If it were practical, for the sake of coherence and consistency, all measurements of a given type in a country would be made using the same national standard in every laboratory in which the measurements are made. However, this is clearly impractical because of the volume of measurements. When industrial measurements are made, it is critical that each be made with accuracy sufficient for its intended use. One way to ensure this is to establish the relationship of the result of a measurement made using an industrial instrument with that which would have been obtained using the corresponding national standard. In a real sense the goal is “accurate” measurement, that is measurement deviating with acceptable uncertainty from a recognized standard, and traceability is a part of reaching that goal.

The formalism of traceability is the tool that provides these measurement relationships. It is the process by which acceptable measurements with well-understood uncertainties can be documented to the degree required by interested parties. At its root, the primary use of traceability is to answer the questions (of auditors, regulators, those with a need for the “right” answer, …): “What correction should be applied to a measurement result obtained at a given time with my instrument to match the result that would be obtained using the instrument (standard) to which traceability is desired? What is the uncertainty of this corrected measurement result?”

It should be noted that the result of a measurement, and its traceability, may be useful even when the measurement uncertainty is relatively large. It is for the user of the measurement to state the allowable magnitude of uncertainty for specific measurement applications.

### 1.2 Current Definitions of Traceability

Probably the most widely-used and accepted definition of traceability is given in the 1993 *International Vocabulary of Basic and General Terms in Metrology* (VIM) [1], published by ISO, as: “property of the result of a measurement or the value of a standard whereby it can be related to stated references, usually national or international standards, through an unbroken chain of comparisons all having stated uncertainties.” There are variations of this definition, e.g., Refs. [3, 4, 5], that introduce the important additional requirement that quality assurance systems be in place. However, while possibly implied, there seems to have been no formal explicit statement of the need to consider the role that time plays in the definition of traceability.

### 1.3 New Definitions of Traceability

In his presentation at the 5th United States-Italy Bilateral Seminar [6], Dr. Robert Hebner, the Acting Deputy Director of NIST, presented the following definition of traceability: “The property of the result of a measurement or the value of a standard whereby it can be related to stated references, usually national or international standards, through an unbroken chain of comparisons all having stated uncertainties. It is noted that traceability only exists when scientifically rigorous evidence is collected on a continuing basis showing that the measurement is producing documented results for which the total measurement uncertainty is quantified.” Note that the first sentence repeats the VIM definition. The second sentence is new, and was meant to emphasize that a single measurement result is sufficient to establish uncertainty relationships only over a limited time interval, and that direct periodic comparisons are otherwise required. In accordance with this principle, we show here that internal measurement assurance, using control (check) standards [1], is required to fully demonstrate that uncertainties remain within stated, acceptable levels when establishing long-term traceability relationships. An uncertainty cannot be stated rigorously without demonstrated traceability. The rigor and level of detail of the measurement assurance procedures required for traceability necessarily depend on the relative levels of uncertainty of the standards involved.

The next section details how such traceability can be achieved. In particular, it shows why it is important to explicitly include the timeline of all relevant measurement events (the “metrological timeline”) that supports and constitutes the chain of comparisons in a “statement of traceability.”

Note that in these definitions, it is the result of a measurement or value of a standard that possesses traceability. Strictly speaking, then, traceability is *not* a property of an instrument or a laboratory, but is a property of the outcome of a process which involves instruments and laboratories. Such shorthand designations are frequently used, however, and so it is important in such cases to specify the range of operation covered by the instrument, or the applicable metrological variables and ranges for the laboratory, for which the traceable condition applies. The fact that traceability is a property of the result of a measurement, and not of an instrument or a laboratory, emphasizes that each measurement result has its own associated uncertainty specific to the circumstances in which it was obtained.

## 2. Metrological Timelines

The most important aspect of a measurement process is to “get the measurement right” to some level. However, defining what “getting the measurement right” means is not always clear or straightforward. This is especially true if what is being measured (the measurand) is changing significantly with time, if the instrument being used to make the measurement is changing significantly with time (e.g., drifting), or if the reference standard to which the measurement is to be traceable is changing significantly with time. A rigorous, comprehensive statement of traceability must be capable of defining the measurement process and the associated measurement uncertainties clearly enough that the relatively “instantaneous” measurement result can be “gotten right,” even when things change with time, as they frequently do. The use of metrological timelines greatly facilitates dealing with time variations of the measured values of quantities that are presumed to be stable when documenting traceability.

### 2.1 Measurement Uncertainty

Assessing measurement uncertainty is at the core of establishing traceability of a measurement result, and so it is important to have an accepted, well-established technique for assessing measurement uncertainty under a variety of measurement conditions. The 1995 *Guide to the Expression of Uncertainty in Measurement* [7], published by ISO, and the 1994 Edition of NIST Technical Note 1297 [8] address the questions of how to assess and express measurement uncertainty, especially if what is being measured is not changing with time. In its scope, the *Guide* states that it “is primarily concerned with the expression of uncertainty in the measurement of a well-defined physical quantity—the measurand—that can be characterized by an essentially unique value.” To demonstrate traceability, the principles of the *Guide* can be used to assess individual uncertainties at “discrete measurement events,” defined here as those covering time periods that are relatively short in comparison with the time period over which the measurand might change. For any case where there is a known, time-dependent systematic error (such as a documented drift over time in the use of an instrument), a simple way of incorporating such a known, uncorrected error into uncertainty considerations has been suggested by Phillips, Eberhardt, and Parry [9]. Note that the *Guide* recommends correcting for such systematic errors whenever possible, and including a component of uncertainty for this correction.

What follows in this section is a description of the key elements of measurement assurance systems and metrological timelines used to develop rigorous yet practical statements of traceability. The measurement assurance system in an NMI is used as an example to develop the concept of the metrological timeline. Monitoring the stability of the national standards is essential, but those standards are usually maintained at a level sufficient to minimize the concerns of those wishing to demonstrate traceability to national standards. Thus it is not ordinarily necessary to take into account the stability of the national standards when demonstrating traceability from lower levels. Only in rare cases, where extremely small uncertainties are required, such as development of a new International Temperature Scale, is it necessary to correct for such changes. Laboratories below the national level need to document the measurement assurance they use to demonstrate traceability from their levels to the national level, just as the NMIs need to document the measurement assurance they use to demonstrate traceability to standards representing the SI system of units. The techniques cited here for the national level can also be used to demonstrate traceability at lower levels.

Clearly those measurement applications that permit larger uncertainties can be supported with less attention to the documentation of measurement quality. By reducing uncertainties beyond immediate levels of need, NMIs bring overall economies to their fields of measurement: investments by the NMI produce benefits which are widely distributed across the user community in terms of ease of use and reduced field-level costs.

### 2.2 Metrological Timelines for Measurement Assurance

**Fig. 1:**
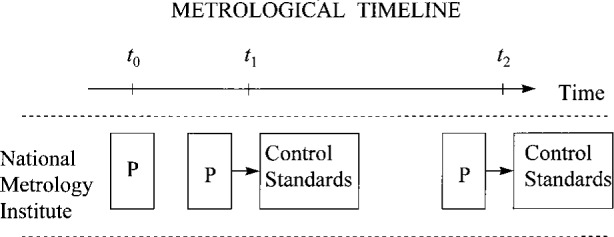
Simple metrological timeline indicating three metrological “events” in a National Metrology Institute.

Figure 1 is a simple metrological timeline, illustrating one of several possible internal measurement assurance systems for a primary measurement standard (denoted by the box containing the letter P) in an NMI. The time axis is shown along the top of the figure, with time increasing from left to right. Three “metrological events” are indicated on the time axis (by the ticks at times *t*_0_, *t*_1_, and *t*_2_). The time axis is not to scale, but rather depicts schematically the sequence of events. Similarly, the “time duration” of an event under any of the ticks, which could be indicated schematically by the width of the set of boxes and arrows “under” a tick, is not to scale. Each event is roughly centered under the appropriate tick on the time axis at which the event takes place. The actual time duration for a particular event will depend on the nature of the event, and supporting documentation can be used to provide such details when necessary.

The first metrological event, represented by the box under the time *t*_0_, occurs at the time that P is first considered available for use. The primary measurement standard P might be a measuring instrument, a reference material, a material measure, or a measuring system [1]. For purposes of this discussion, P will be taken to be the most general type of measurement standard, a measuring system. The same basic principles concerning traceability would apply if P were any other type, but the details might be different. The initial characterization of the primary measurement system P is based on first-principles, “without reference to other standards of the same quantity,” by definition [1]. Such a characterization also involves evaluating the uncertainties associated with using P to make measurements of the quantity that P is designed to measure.

Once the primary measurement system P has been characterized, to be useful in the future its metrological characteristics must be “conserved” [1]. The second metrological event in Fig. 1, represented by the two boxes connected by an arrow under the time *t*_1_, is the calibration of an ensemble of control standards using P. The control standards must be of sufficient quality (stability, repeatability, resolution, etc.) that they can be used to detect changes in behavior or performance of P, using traditional measurement assurance techniques, at a level commensurate with that at which P is to be used. Control standards with such properties can exist even when the standards cannot be characterized from first principles and hence cannot be used as primary measurement standards themselves. Calibrations over time of the ensemble of control standards against P form the basis for a long-term internal measurement assurance system to conserve P.

The third metrological event, represented by the boxes under the time *t*_2_, is a subsequent calibration of the same set of control standards using the same primary measurement system P. By plotting a set of measurement results obtained at times *t*_1_ and *t*_2_, a measurement assurance chart (or a measurement control chart) for the entire system of standards is begun. By repeating the same measurements and monitoring the variation in the measurement results over time, an estimate of the long term stability and repeatability of the system, and in particular of P, can be formulated. This entire system of standards can also be used at some later point in time to validate the immediate operational integrity of P. As shown below, the stability and repeatability of the entire system of standards become important components of uncertainty in a final statement of traceability. Note that the measurement assurance method described here may not always be applicable to a primary measurement system, but related methods that accomplish the goal of monitoring the integrity of the system over time are typically developed and used.

### 2.3 Simple Metrological Timelines for Traceability

Figure 2a illustrates a slightly more complicated metrological system than that in Fig. 1. In this case, a measurement artifact is shown explicitly as part of the internal measurement assurance system used to monitor the stability of P. The measurement artifact (or “material measure” [1]), belonging to the NMI, is a “device intended to reproduce or supply, in a permanent manner during its use, one or more known values of a given quantity.” This timeline introduces the measurement artifact to provide explicit reference to measurement results and measurement uncertainties. As indicated at time *t*_1_, P is used to perform a measurement of the quantity *X* on the measurement artifact, with the measurement result $X_{1}^{P}$ having uncertainty [7, 8] $U_{1}^{P}$. At about the same time *t*_1_, the ensemble of control standards (N*_i_*, where *i* is an index representing the number of control standards in the ensemble) is used to make measurements of the same quantity *X*, with measurement results $X_{1}^{N_{i}}$ having uncertainties $U_{1}^{N_{i}}$. Similarly, at time *t*_2_, P is again used to perform a measurement of the same quantity *X* on the same measurement artifact, with the measurement result $X_{2}^{P}$ having uncertainty $U_{2}^{P}$, and the same set of control standards (N*_i_*) are used to make measurements of the quantity *X*, with measurement results $X_{2}^{N_{i}}$ having uncertainties $U_{2}^{N_{i}}$.

An illustrative chart of the type described above is presented in Fig. 3a. This simple chart records measurement results (*X*, indicated by the dots) and uncertainties (*U*, indicated by the error bars) for the primary measurement system P and two different control standards (N_1_ and N_2_) at the two times *t*_1_ and *t*_2_. Such data demonstrate the functional integrity of the full system of instruments (which includes P, the control standards, and the measurement artifact), within the scatter of the data and their uncertainties, over the time period covered by the chart. Note that in order for such a system to work effectively, the designs of the control standards should differ from each other and from P, thus reducing the possibility that changes in individual performance over time are correlated with one another, or with changes in the primary measurement system. In many cases a single control standard would be sufficient to demonstrate whether or not the measurement system is under control. Also note that while the simple, illustrative chart in Fig. 3a contains data for only two times, actual charts would include many data sets, thus allowing long-term monitoring of the system.

While the example in Fig. 2a and Fig. 3a is given for a primary measurement standard in an NMI, there is also a need for the same type of internal measurement assurance system for the standards used by other laboratories, such as a calibration laboratory that is a customer of the NMI, as indicated in Fig. 2b, and the control chart in Fig. 3b.

Note that while traceability is usually regarded as relating measurement results to specific standards (e.g., instruments or systems), the relationship must also include all aspects of the measurement process (including influence quantities such as environmental conditions) that effect the overall measurement uncertainty. Later in this paper, the shorthand notation of describing a measurement result as traceable to a standard implies that uncertainties for the entire measurement process (under specified conditions) are included.

The simple example in Fig. 2b includes an aspect of traceability that should always be considered: the possible time dependence of the performance of the calibration laboratory’s reference standard C. Strictly speaking, the measurement result $X_{2}^{C}$ is traceable to the standard C as it existed at the time *t*_2_, but not necessarily as it existed at the time *t*_1_. To exemplify why such distinction is necessary, suppose that in the period between times *t*_1_ and *t*_2_ the reference standard C was somehow modified (e.g., damaged), exhibited drift, or the uncertainty associated with its use was somehow changed, then the measurement result $X_{2}^{C}$ and the uncertainty $U_{2}^{C}$ might not be related to the state of C at time *t*_1_ in a known or well-understood way. Under such circumstances, it would not be reasonable to claim traceability of the measurement result $X_{2}^{C}$ to C as it existed at the time *t*_1_.

Data of the kind shown in Fig. 3c suggest that the performance of the reference standard C had changed between times *t*_1_ and *t*_2_. That is, the value of the measurement result $X_{2}^{C}$ obtained at time *t*_2_ is significantly above the values of all of the other measurement results. In this case, C has shifted by an amount $\delta X^{C}=X_{2}^{C}-X_{1}^{C}$. In order to claim traceability of the measurement result $X_{2}^{C}$ obtained at time *t*_2_ to the reference standard C as it existed at time *t*_1_, the calibration laboratory would have to incorporate $\delta X^{C}$, preferably as a correction to the measured value in the statement of traceability, or *as an additional component of uncertainty* $\left(\delta U^{C}\right)$ *associated with the statement of traceability*. Note that if the calibration laboratory did not perform measurements of the type displayed in Fig. 3c, the change in performance of C would remain unknown, and subsequent claims of measurement values or uncertainties in statements of traceability would be in error.

### 2.4 More Complex Metrological Timelines for Traceability

**Fig. 4:**
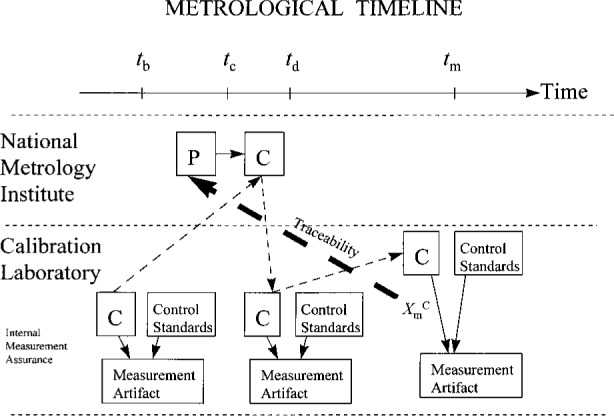
Metrological timeline demonstrating the traceability of a measurement result in a Calibration Laboratory to a standard in a National Metrology Institute.

Figure 4 is a metrological timeline depicting the metrological events relevant to the traceability of a measurement performed by a calibration laboratory to a standard in an NMI. As indicated in the figure, the calibration laboratory seeks to establish traceability of a measurement result $X_{m}^{C}$, obtained while using the calibration laboratory’s reference standard C at the time *t*_m_, to the primary measurement system P. Also indicated in the figure are metrological events relevant to the traceability of the measurement result obtained at time *t*_m_ recorded at the earlier times *t*_b_, *t*_c_ and *t*_d_: time *t*_c_ is the time when the NMI calibrates C using P: time *t*_b_ is a time prior to time *t*_c_ when C has been characterized in the calibration laboratory as part of an internal measurement assurance process that incorporates the calibration laboratory’s measurement artifact and control standards, as described above: time *t*_d_ is a time after C has been returned by the NMI to the calibration laboratory. Time *t*_d_ is the time when C is used as part of the internal measurement assurance system in the calibration laboratory, in a manner identical (including its initial characterization) with that used at time *t*_b_.

The steps at times *t*_b_ and *t*_d_ are both needed to establish confidence in the integrity of the traceability statement. These steps allow the calibration laboratory to verify that C is not damaged or otherwise adversely affected beyond acceptable limits during its journey to and from the NMI. The verification is accomplished by comparing results (e.g., with control charts described earlier) obtained when using C in an identical manner at times *t*_b_ and *t*_d_. The thin dashed arrows in the figure are to aid in following the sequential use of C along the timeline.

In Fig. 4, the traceability is indicated schematically by the heavy arrow, which relates the measurement result $X_{m}^{C}$, obtained at time *t*_m_, to the standard P as it existed at the time *t*_c_. For simplicity in the figure, the traceable measurement result $X_{m}^{C}$ is shown being obtained during the course of taking data for internal measurement assurance purposes. If, instead, the reference standard C had been used to perform some other measurement at time *t*_m_, for which traceability to P was desired, then it would have been important to perform yet another internal measurement assurance operation (i.e., taking more data) afterwards, using the same measurement artifact and control standards. This last step would verify that C was still performing within acceptable limits after the traceable measurement at time *t*_m_, and hence was likely to do so at time *t*_m_ as well.

**Fig. 5:**
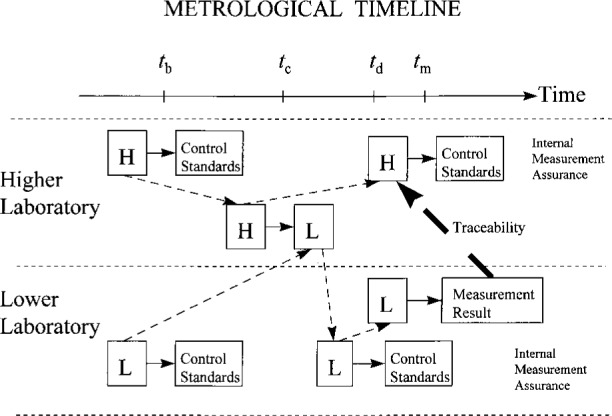
Metrological timeline demonstrating the traceability of a measurement result obtained in a laboratory lower in a traceability hierarchy to a standard maintained in a laboratory higher in the traceability hierarchy.

**Fig. 6:**
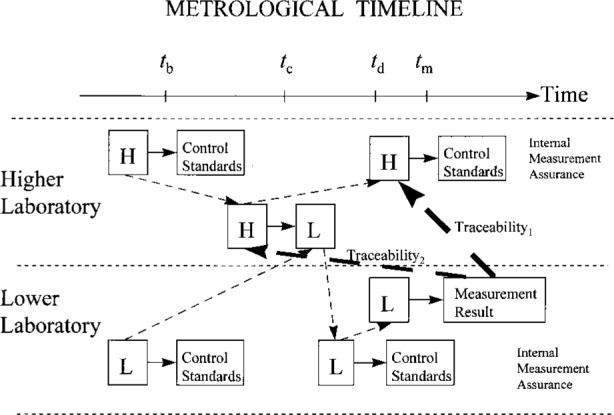
Metrological timeline demonstrating two possible traceability paths for a measurement result obtained in a laboratory lower in a traceability hierarchy to a standard maintained in a laboratory higher in the traceability hierarchy.

A metrological timeline highlights the key elements of a traceability relationship. For more complicated relationships, the utility of a metrological timeline becomes even more apparent, as illustrated in Fig. 5. This figure depicts a laboratory lower in a traceability hierarchy sending its reference standard L to a higher laboratory for calibration against that laboratory’s reference standard H. The measurement assurance systems of both laboratories are explicitly indicated, although, for simplicity, explicit use of measurement artifacts in both laboratories is suppressed in the figure. Depending upon the likelihood that the calibration of H has changed significantly between times *t*_c_ and *t*_m_, for reasons discussed earlier, the lower laboratory might wish to demonstrate traceability of the measurement result to H as it existed at the time *t*_m_ (indicated schematically by the heavy arrow). However, the lower laboratory may also desire a traceability statement relating the measurement result to H as it existed at time *t*_c_, since that is when the lower laboratory’s reference standard L was actually calibrated against H. Either of these traceability statements is possible; however, in general, the measurement values and the uncertainties associated with the traceability statements will differ. Having a metrological timeline like that shown in Fig. 6, where both traceability paths are indicated on the same page (as Traceability_1_ and Traceability_2_), would greatly assist a measurement auditor to visualize the difference between the alternative traceability statements.

## 3. Statements of Traceability

As discussed in the introduction, the use of traceability is primarily to answer the questions: “What correction should be applied to a measurement result obtained at a given time with my instrument to match the result that would be obtained using the instrument (standard) to which traceability is desired? What is the uncertainty of this corrected measurement result?”

The examples given in earlier sections demonstrate how the clear and rigorous identifications of metrological events and measurement relationships help, for even the simplest traceability statements, to answer these questions. To demonstrate the unbroken chain of measurement and uncertainty relationships between the measurement for which traceability is claimed and the standard to which traceability is claimed all the relevant parameters must be defined and described. Metrological timelines, control charts, and records detailing all relevant metrological parameters associated with use of the instruments or standards involved at each step along the way are important tools which make it possible to describe unambiguously how the uncertainty associated with the measurement has been evaluated.

On the practical side, while it is important to address the issues discussed above, the degree to which measurements and relationships must be documented may vary considerably, in part based on considerations of cost. For instance, less attention can be given to a standard’s instability if the changes are small when compared to the uncertainty of the measurement. However, it is not wise to ignore the performance of the reference standard, since it too may undergo a serious change in performance: it may, for example, be damaged.

### 3.1 Components of a Practical, Rigorous Statement of “Simple” Traceability

While practical considerations cannot be disregarded, it is useful for completeness, and to make sure that nothing has been overlooked, to list all the metrological events that must be considered in documenting the traceability of a measurement result. The key elements of a general statement of traceability for the relatively simple examples presented above are:

1. Provision of a complete metrological timeline (similar to those in Figs. 4–6, with accompanying descriptive text) identifying all relevant physical components, including control standards, and measures used to demonstrate the traceability of the measurement result and the integrity of its uncertainty. Note: It is useful to document how control standards are used to ensure that no instruments or systems, especially those that are moved or transported, experience a significant unpredicted change in their performance over time. In particular it must be demonstrated, using the same control standards both before and after an instrument or system is shipped, that it performs in essentially the same manner after it is received as before it was shipped. For completeness, acceptable performance of the instrument or system should be verified following a measurement for which traceability is being demonstrated. Usually, this is done by comparison with control standards, documenting the procedure and results.
2. Description of all of the metrological details associated with the measurement, including what was measured, the result of the measurement, the instrument(s) or system(s) used, when and where the measurement took place, the measurement environment, the results of all ancillary measurements and their estimated uncertainties, who performed and who was responsible for the measurement, what calculations, models or analyses were used to obtain the measurement result, and the uncertainty of the measurement result.
3. Definition of the standard to which the measurement result is to be traceable, including the point in time in the existence of the standard at which the traceability is being established, and all metrological details (as described in 2 above) of the standard that influence the uncertainty of the measurement result. Note: If the uncertainty, and any change in uncertainty, associated with the standard is much smaller than the uncertainty of the measurement result for which traceability is being documented, then only a brief description of the measurement history of the standard is needed as long as the standard performed within expected limits when the lower laboratory’s standard was compared to it.
4. Provision of the uncertainty analysis including supporting documentation, such as test results and control charts, used to calculate the uncertainty of the measurement result for which traceability is being demonstrated. If there is an “additional” component of uncertainty (such as *δU*^C^ above) associated with the point in time that the traceability to a higher-level standard is being established, this uncertainty must be clearly identified, and its incorporation into the overall statement of uncertainty described.

As noted above, depending on specific requirements, statements of traceability need not include all of these components. However, if a statement of traceability omits one or more of the components, it should be noted in the statement what components have been omitted, and why. In general, there is no such thing as “partial traceability.” If any aspect of the measurement chain is not given due consideration, the reported uncertainty of the measurement result in the statement of traceability is inherently suspect.

### 3.2 Components of a Practical, Rigorous Statement of More Complex Traceability

**Fig. 7:**
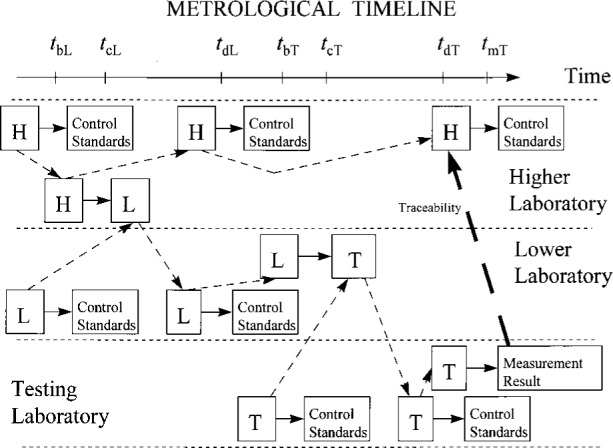
Metrological timeline demonstrating traceability of a measurement result obtained in a testing laboratory to a standard maintained in a laboratory higher in the traceability hierarchy, through a laboratory intermediate in the traceability hierarchy.

The principles of measurement traceability discussed thus far can be applied fairly straightforwardly to situations where the measurement result is traceable to the desired standard through intermediate standards. The added complexity gives rise to several new issues. Perhaps the most important of these has to do with responsibilities of record keeping and providing information, as can be demonstrated with the aid of the metrological timeline shown in Fig. 7. Pictorially, in the center of this figure, a testing laboratory obtains a calibration of its standard T from the lower laboratory at time *t*_cT_. The testing laboratory subsequently performs a measurement using the standard T at time *t*_mT_ and wishes to demonstrate traceability of that measurement result to the standard H of the higher laboratory.

In order for the testing laboratory to assess the measurement uncertainty associated with the measurement result at time *t*_mT_, the uncertainty $U_{t_{\mathrm{mT}}}^{T}$ associated with the standard T at time *t*_mT_ must be known. This latter uncertainty can be evaluated in the testing laboratory from the control charts maintained on the standard T during the time period from *t*_cT_, when the standard T was calibrated by the lower laboratory’s standard L and then returned to the testing laboratory, to the time *t*_mT_. If the time-average of the measurement quantity *X*^T^ (denoted $X_{\mathrm{ave}}^{T}$), used in the control chart as a control parameter, changes (e.g., drifts) by an amount $\delta X_{\mathrm{ave}}^{T}$ between *t*_cT_ and *t*_mT_, then the testing laboratory can either “adjust” the values assigned to measurements made using T, or adjust the uncertainty assigned to T [9]. The original uncertainty associated with the standard T at the time *t*_cT_ can be ascertained in the testing laboratory from the calibration report corresponding to calibration of the standard T against the lower laboratory’s standard L at the time *t*_cT_. This calibration report should be provided to the testing laboratory from the calibration laboratory around the time *t*_cT_. The method used to incorporate $\delta X_{\mathrm{ave}}^{T}$ into the uncertainty of the measurement result for which traceability is being established needs to be specified [9].

The uncertainty associated with the lower laboratory’s standard L at the time *t*_cT_ can be assessed in a similar manner by the lower laboratory. This uncertainty can be estimated from the control charts maintained on the standard L during the time period from *t*_cL_, when the standard L was calibrated against the higher laboratory’s reference standard H and then returned to the lower laboratory, to the time *t*_cT_. The original uncertainty associated with the standard L at the time *t*_cL_ can be derived from the calibration report, issued by the higher laboratory, corresponding to calibration of the standard L against the standard H at the time *t*_cL_. As in the case for the testing laboratory, if the time-average of the measurement quantity *X*^L^ (denoted $X_{\mathrm{ave}}^{L}$), used in the control chart as a control parameter, changes by an amount $\delta X_{\mathrm{ave}}^{L}$ between *t*_cL_ and *t*_cT_, then the lower laboratory can either adjust the values assigned to measurements made using L, or adjust the uncertainty assigned to L, when it is used to calibrate T.

A comprehensive statement of traceability of the measurement result at the time *t*_mT_ thus requires, in principle, documentation of the performance history of the test standard T, the lower laboratory’s standard L, and the higher laboratory’s standard H. If the traceability of the measurement result is to be to H as it exists at the time *t*_mT_, then a quantity $\delta X_{\mathrm{ave}}^{H}$ will be needed as a correction factor or as an additional component of uncertainty associated with the measurement result. The definition of $\delta X_{\mathrm{ave}}^{H}$ is, as above, the change in the time-average of the measurement quantity *X*^H^ (denoted $X_{\mathrm{ave}}^{H}$), used in the control chart as a control parameter, between the times *t*_cL_ and *t*_mT_.

The testing laboratory’s difficulty in assembling a comprehensive statement of traceability is then twofold. First, the lower laboratory does not typically provide the relevant performance history of the standard L in its calibration report, but only the uncertainty associated with the standard L at the time *t*_cT_. However, better laboratories will be able to provide such information, covering a reasonable period of time, upon request. Second, for a system of standards only slightly more complex than that indicated in Fig. 7, or for traceability paths covering a span of several years, it could prove difficult to identify the standard H in the higher laboratory, especially if it has undergone modification, and harder yet to obtain access to the quantity $\delta X_{\mathrm{ave}}^{H}$, thus adding to the difficulty of assembling a comprehensive statement of traceability.

Fortunately, in many cases requiring traceability, these issues do not materialize due to the increasing levels of acceptable uncertainty as one descends the laboratory hierarchy. For example, if the testing laboratory in Fig. 7 only requires modest uncertainties when using the reference standard T, and the variations in the reference standards in the laboratories higher in the hierarchy are significantly below this uncertainty over the time period, then the testing laboratory can exclude these variations from the uncertainty analysis. However, if either $\delta X_{\mathrm{ave}}^{H}$or $\delta X_{\mathrm{ave}}^{H}$ are similar in magnitude to the uncertainty associated with using T, then they must be applied as a correction or incorporated into the final uncertainty of the measurement result using T at time *t*_mT_ in the statement of traceability. In either case the traceability analysis needs to show that this issue has been addressed. It is clear that when higher laboratories establish sufficiently small uncertainties, uncertainty analyses in lower laboratories are simplified and record keeping may be reduced.

The hierarchy of measurement and testing laboratories (national laboratories, secondary laboratories, and testing laboratories, etc.) has been established in such a way that, ordinarily, a laboratory need only go to one level up to obtain the necessary calibration uncertainty, but usually will need to have traceability to more than one level up. For instance, in the example of Fig. 7, the testing laboratory should only have to go to the lower laboratory to obtain the needed calibration uncertainty, but will have to obtain information from the higher laboratories to have traceability to national standards.

If the testing laboratory had needed to obtain a smaller uncertainty than that obtained from the lower laboratory, however, it could have sent its standard T directly to the higher laboratory for direct calibration against the standard H shortly before the measurement result required at the time *t*_mT_ was obtained. This is frequently not possible for reasons of cost and time. When possible, however, such a procedure would allow the testing laboratory to assess how closely the measurement results agree for the two traceability paths, as well as how realistically the uncertainty evaluation of the measurement result at the time *t*_mT_ was carried out using the original traceability path.

## 4. Other Issues Concerning Statements of Traceability

### 4.1 Traceability When More Than One Standard is Used for a Calibration

Sometimes a lower laboratory’s measurement standard L sent to a higher laboratory for calibration requires that more than one reference standard H be used by the higher laboratory to perform the calibration adequately. The reference standard H is taken here to be a measuring instrument. Such is the case, for example, when the operating range of L does not overlap well with any of the standards H in the higher laboratory. Ehrlich, Eberhardt, et al. [10] have described a statistical algorithm for deriving the measurement uncertainty in such a situation for pressure standards. Under these circumstances the traceability of a measurement result is not to an individual instrument, but rather to a complex measurement system whose elements are the measurement instruments used, and the program of analysis is used to derive the uncertainty statement in the calibration report associated with the lower laboratory’s standard L. Care must be taken to document the historical data (e.g., using control charts) associated with these measurement systems so that traceability statements may be attached to measurements based on subsequent use of the lower laboratory’s reference standard L.

### 4.2 Intrinsic Standards and Traceability

Another complex issue concerning traceability is the use of intrinsic standards. The key question is whether or not intrinsic standards can serve as stand-alone, turnkey systems requiring no prior comparisons with other standards while still providing inherent traceability to the other standards. Intrinsic standards differ from ordinary transfer standards in that they may be characterized in much the same way as primary standards. The *ANSI/NCSL Z540-1-1994, American National Standard for Calibration—Calibration Laboratories and Measuring and Test Equipment—General requirements* [11], states that intrinsic standards are “based on well-characterized laws of physics, fundamental constants of nature, or invariant properties of materials and make ideal stable, precise, and accurate measurement standards if properly designed, characterized, operated, monitored, and maintained.” However, the ANSI/NCSL Z540-1 goes on to state: “Where intrinsic standards are used, the laboratory should demonstrate by measurement assurance techniques, interlaboratory comparisons, or other suitable means that its intrinsic standard measurement results are correlated with those of national or international standards.” The National Conference of Standards Laboratories, at its July 1997 meeting [12], readdressed the issue of describing intrinsic standards and provided the following draft definition:

Intrinsic (measurement) standard:

Standard recognized as having or realizing, under its prescribed conditions of use and intended application, an assigned value the basis of which is an inherent physical constant or an inherent and sufficiently stable physical property.

Notes:

1. An intrinsic standard usually consists of a device or system based on the requirements of a documented, consensus method.
2. The value of an intrinsic standard is assigned by consensus and does not need to be established by calibration or comparison with another standard. Its uncertainty is determined by considering two components: (a) that associated with its consensus value and (b) that associated with its construction and implementation.
3. To establish and ensure stability and/or traceability, the value of an intrinsic standard and the uncertainty associated with its construction and implementation should be verified at appropriate intervals. Verification may be carried out either by applying a recognized, consensus test method or by intercomparisons among comparable standards. Such intercomparisons may be accomplished with standards in a local quality control system or with external standards including national and international standards.

The debate continues as to whether or not measurement results using intrinsic standards can be considered traceable to other measurement standards without benefit of direct intercomparison.

### 4.3 Multiple Routes to Traceability

When a calibration laboratory requires direct traceability of a measurement result to a reference standard in an NMI for one of the SI base quantities (mass, length, time, etc.), the calibration laboratory must go directly to the organization in the NMI that provides appropriate measurement services. But if a calibration laboratory requires direct traceability to an NMI for a measurement result of a derived quantity (e.g., pressure), then that laboratory can select from two options based on practicality. One is to have a standard calibrated against a reference standard for the derived quantity maintained at the NMI. The second is to obtain calibrations from the NMI for all the base quantities needed to develop a primary standard for the derived quantity. In the second case, metrological timelines must be developed and maintained for each of the base quantities.

Elaborating further, the first, most straightforward option, is to go to the organization in the NMI that provides measurement services for the derived quantity. The organization provides this capability by developing primary standards based on measurement results that are themselves traceable to reference standards, maintained by other organizations in the NMI, that are representations of the SI base units. These representations are in turn based on realizations of the definitions of these units. Note that, while traceability to a reference standard that is a representation of an SI unit is possible, it is not appropriate to describe measurements as traceable to the SI unit itself, except possibly to the unit of mass, for which the SI unit is defined in terms of an international prototype. The preferred form is to describe a measurement result as “expressible” in terms of SI units [13].

The second option is to characterize a primary standard at the calibration laboratory by obtaining calibration services for the base quantities directly from the NMI. Alternatively, the calibration laboratory could obtain a primary standard characterized by another laboratory using calibration services obtained directly from the organizations in the NMI that develop and characterize reference standards that are representations of the SI base units. Examples of both options can be found in practice.

While these options may both satisfy the requirement that a measurement result be directly traceable to an NMI, it is important to note that the options may provide final measurement results and associated uncertainties that differ widely. This is because the primary standard developed by the calibration laboratory may be different from that developed by the NMI. Even if these standards are of identical design, minor differences in the implementations or models used for assessment of the final measurement results may change the values and uncertainties ascribed to the standards.

The use of comprehensive statements of traceability that contain metrological timelines of the types described above should provide ample opportunity to understand which traceability path is being used by the calibration laboratory, and what assumptions are being made.

### 4.4 Recalibration Intervals

Related to the issue of traceability is the question of the frequency with which instruments should be recalibrated. Natural wear on an instrument due to the “hardness” and frequency of use, mishandling, environmental factors such as corrosion, and even the way an instrument is used, all have a bearing on instrument performance. A significant change in instrument performance, or an uncertainty not within desirable limits, will typically warrant recalibration of the instrument. However, as discussed at length in Ref. [14], the cost of frequent recalibration balances the economic (and other) advantages of keeping an instrument within desirable limits of uncertainty.

An appropriate recalibration interval can be established by reference to the control chart which results from routine comparison with control standards. Furthermore, abnormalities can be quickly spotted and corrected by means of repair and recalibration. Determining when to recalibrate an instrument by using control standards usually results in less frequent recalibrations, saving time and money. Of course, the cost of purchasing and/or developing, using and maintaining control standards must be considered in the cost accounting, but when traceability is required, the control standards must be used and become part of the cost of doing business anyway.

## 5. Considerations at the International Level

The explicit examples presented above using metrological timelines to portray the unbroken chain of comparisons in traceability relationships apply specifically within a nation. In the growing global economy, the questions posed earlier at the root of domestic traceability requirements, namely, “What correction should be applied to a measurement result obtained at a given time with my instrument to match the result that would be obtained using the instrument (standard) to which traceability is desired? What is the uncertainty of this corrected measurement result?” also apply to measurement relationships that cross national boundaries. That is, someone performing a measurement in one country may need to know or demonstrate how the result of that measurement relates to what the result of that measurement would be if carried out by the NMI (or a calibration laboratory) of another country. While the concepts of traceability discussed above may sometimes be applicable to this situation, this is frequently not the case since NMIs do not typically calibrate each other’s instruments, but rather compare measurement capabilities through international intercomparisons or round robins using intermediate or transfer standards. Depending upon the details, results from such intercomparisons could sometimes be used to establish traceability to standards of another nation.

It is becoming more common to hear the term “equivalence” used to describe the metrological relationship when national boundaries are crossed. In these cases, the measurement capabilities of the various NMIs have been demonstrated to be equivalent within a certain range. The term traceability is being reserved for the more common usage of domestic traceability described in the main text above. The term equivalency, then, links measurements or standards at the same level. There is no hierarchical relationship between equivalent measurements or standards. Traceability, on the other hand, links measurements or standards through a hierarchical chain. Each link in the chain is either above or below the one next to it, even if they are in the same organization. These two terms signify very different concepts [15].

A point mentioned earlier, but worth reemphasizing here, is that in the VIM definition traceability is the property of the result of a measurement or the value of a standard, but not the property of an instrument or a laboratory. Thus, within this conventional definition, it is not proper to think of an NMI being traceable to another NMI, even though measurement results could, in some cases, be established as traceable to standards in another NMI. From this perspective also, the term “equivalence” of national laboratories in particular metrological areas is preferred.

As noted earlier, it is not within the scope of this paper to develop a formalism using metrological timelines for describing the metrological relationships corresponding to traceability across national boundaries. To minimize confusion, words other than traceability should probably be used to describe the relationship between the result of a measurement in one country’s NMI and the corresponding (hypothetical) result of the same measurement carried out by the NMI of another country if a direct link of calibrations (or equivalent for chemical or other standards) cannot be demonstrated. The International Bureau of Weights and Measures (BIPM) is in the process of developing a mechanism by which the abilities of nations to perform nominally identical measurements can be compared and the results published [16] as a basis for establishing equivalence for use in developing mutual recognition agreements.

## Figures and Tables

**Fig. 2a:**
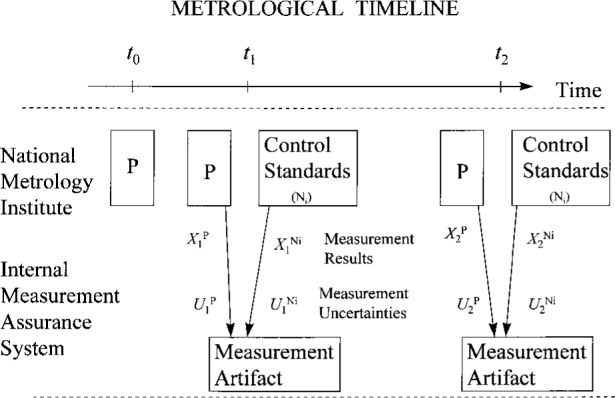
Metrological timeline explicitly detailing an internal measurement assurance system in a National Metrology Institute.

**Fig. 2b:**
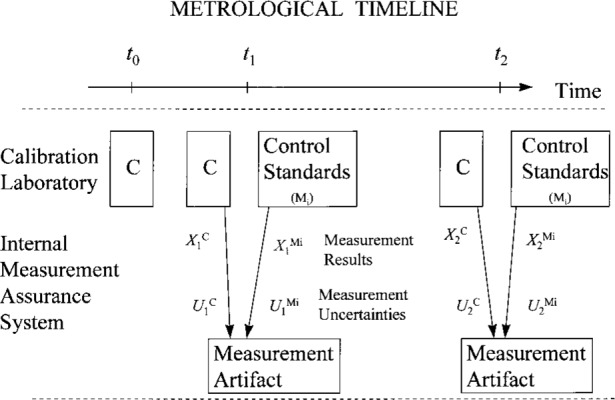
Metrological timeline explicitly detailing an internal measurement assurance system in a Calibration Laboratory.

**Fig. 3a:**
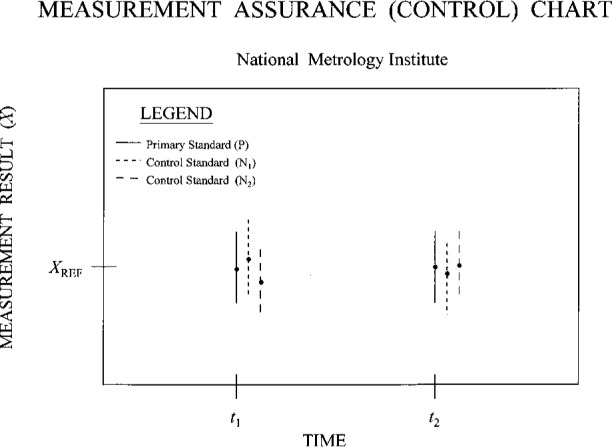
Measurement assurance (control) chart containing sample data taken at the times *t*_1_ and *t*_2_ according to the metrological timeline of Fig. 2a.

**Fig. 3b:**
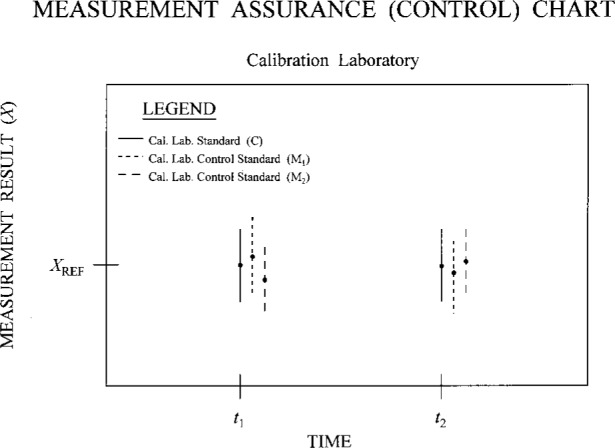
Measurement assurance (control) chart containing sample data taken at the times *t*_1_ and *t*_2_ according to the metrological timeline of Fig. 2b.

**Fig. 3c:**
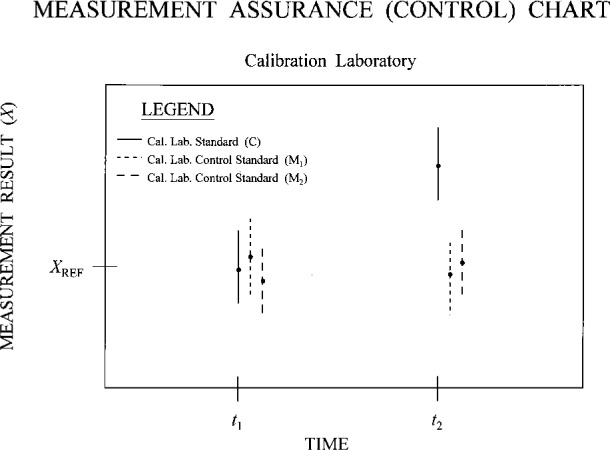
Measurement assurance (control) chart containing sample data illustrating a shift in performance of the calibration laboratory standard C at time *t*_2_ from its performance at time *t*_1_.
